# A New Multi-Parametric MRI-Based Scoring System for Degenerative Cervical Myelopathy: The Severity on Imaging Myelopathy Score (SIMS)

**DOI:** 10.3390/brainsci15060557

**Published:** 2025-05-23

**Authors:** Alexis Morgado, Julien Berthiller, Fabien Subtil, Donato Creatura, Gildas Patet, Nathalie André-Obadia, Cédric Yves Barrey

**Affiliations:** 1 Department of Spine and Spinal Cord Surgery, Hôpital Pierre Wertheimer, Hospices Civils de Lyon, Claude Bernard University of Lyon 1, 59 Boulevard Pinel, 69003 Lyon, France; cedric.barrey@chu-lyon.fr; 2 Service de Biostatistique, Hospices Civils de Lyon, 69003 Lyon, France; julien.berthiller@chu-lyon.fr (J.B.); fabien.subtil01@chu-lyon.fr (F.S.); 3 Department of Neurosurgery, IRCCS Humanitas Research Hospital, 20089 Milan, Italy; donato.creatura@chu-lyon.fr; 4 Department of Clinical Neurosciences, Division of Neurosurgery, Geneva University Hospitals, 1205 Geneva, Switzerland; gildas.patet@hopitalvs.ch; 5 Neurophysiology & Epilepsy Unit, Neurological Hospital P. Wertheimer, Hospices Civils de Lyon, 59 Boulevard Pinel, 69003 Lyon, France; nathalie.obadia-andre@chu-lyon.fr

**Keywords:** cervical spondylotic myelopathy, MRI, SIMS, increased signal intensity

## Abstract

**Background/Objectives:** Degenerative cervical myelopathy (DCM) is the leading cause of functional disabilities of spinal origin in people over 50 years old. The objective of the present study was to establish a multi-parametric weighted scoring system that is easy to use in daily practice, based on the most significant MRI signs and correlated as strongly as possible with the clinical presentation (mJOA)—we call this system the SIMS or Severity on Imaging Myelopathy Score. **Methods**: Ninety-nine patients who underwent clinical and radiological evaluation by mJOA and MRI between January 2015 and March 2021 were retrospectively included. The variables included in the score were the Fujiwara ratio, the T2-weighted intramedullary hyperintensity, the aspect of the peri-medullary fluid cisterns, the Torg–Pavlov ratio, the local kyphosis and the number of stenotic levels. Each variable was first correlated to the mJOA score for each patient, making it possible to construct the final SIMS at the end, and validate it by comparison with mJOA scores. **Results**: The variables that were significantly correlated with one another were the T2-weighted intramedullary hyperintensity, the reduction in peri-medullary fluid spaces and the number of stenotic levels (*p* < 0.05). Then, points were assigned to each variable according to their relative importance and made it possible to construct the definitive SIMS. The final Spearman correlation coefficient between the SIMS and the mJOA score was −0.747. **Conclusions**: This work showed that this new multi-parametric MRI-based scoring system represents a consistent means to characterize the degree of severity of degenerative cervical myelopathy.

## 1. Introduction

Degenerative cervical myelopathy represents the most frequent cause of functional impairment from medullary origin in adult patients over 50 years of age, may result in a major disability like tetraparesia and is the consequence of degenerative changes in the cervical spine [1]. The clinical presentation of DCM is variable [2]. In order to harmonize the assessment of disability due to DCM, the modified-JOA (mJOA) is now commonly used [2,3,4,5].

MRI represents the gold standard imaging modality for the diagnosis of the disease. Different MRI signs have been reported with variable clinical significance [5,6,7,8,9,10,11].

According to the literature, it seems that one MRI sign alone remains poorly correlated with the clinical presentation. The absence of a clear correlation between the symptoms and MRI findings poses some difficulties to precisely evaluating the disease’s severity and can thus make the decision-making uncomfortable for the clinicians.

In order to help practitioners to accurately assess the severity of the disease on MRI and therefore choose the best treatment option, the objective of the present study was to establish a multi-parametric weighted scoring system that is easy to use in daily practice, is based on the most significant MRI signs and correlates as strongly as possible with the clinical presentation (mJOA)—we call this system the SIMS or Severity on Imaging Myelopathy Score.

## 2. Materials and Methods

### 2.1. Study Design and Population

This is a single-center retrospective analysis of retrospectively collected data from 99 surgical patients with a diagnosis of cervical degenerative stenosis on MRI and who had a clinical evaluation with mJOA score established by at least one of the senior neurosurgeons of the Spine and Cord Unit of the Neurological Hospital of the Hospices Civils de Lyon (HCL), between January 2015 and March 2021, and who benefited from a cervical MRI with axial and sagittal slices, both within an interval of less than 1 year. The indication for surgery was left to the surgeon’s discretion and could be based on the patient’s clinical and functional assessment, radiological evolution or abnormalities found on evoked potentials.

Inclusion criteria were as follows: (1) age ≥18 years; (2) mJOA score found in the patient’s medical record; (3) cervical MRI with T2-weighted sequences including axial and sagittal slices.

The following patients were excluded from the study: patients with (1) age < 18 years; (2) a history of surgery and/or trauma and/or infection and/or neoplasia and/or congenital deformity of the cervical spine; (3) the presence of severe and/or advanced neurological or systemic disease that could influence the clinical or electrophysiological evaluation.

Informed consent was obtained from the participants. The study was approved by the Ethical Committee of French College of Neurosurgery (IRB00011687). All methods were carried out in accordance with relevant guidelines and regulations.

### 2.2. Selection of Variables

The selection of the parameters composing the SIMS was made on the basis of literature searches, on the PubMed platform, with the aim of retaining the variables best correlated with clinical symptoms. These parameters had to be measurable on MRI images using the HCL visualization software (PACS) at the most stenotic level.

The measurement of the different variables for each patient was performed jointly by 2 neurosurgeons specialized in spine surgery (CB and AM). The determination of the highest stenotic level was made according to a consensus between the two practitioners.

Initially, 8 variables were selected on the basis of the literature data. Due to the lack of a clear correlation with the clinical presentation in the statistical analysis of 2 of the criteria, it was decided to keep 6 criteria: the Fujiwara ratio; T2-weighted intramedullary hyperintensity; cerebrospinal fluid cisterns; the Torg–Pavlov ratio; local kyphosis; and the number of stenotic levels.

### 2.3. Categorization for Each SIMS Criteria

#### 2.3.1. Fujiwara Ratio

**Figure 1:**
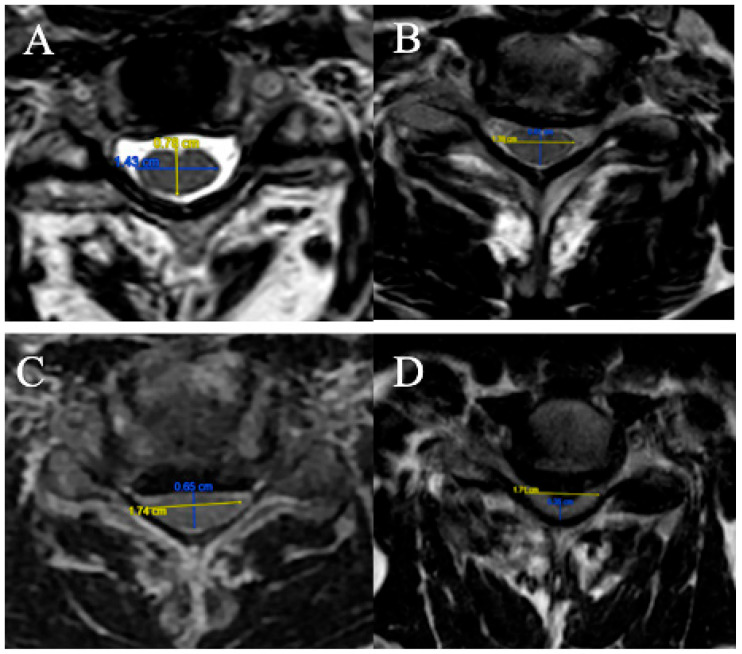
FR measured, respectively, at 0.55 (**A**), 0.44 (**B**), 0.37 (**C**) and 0.20 (**D**)**.**

The FR is defined as the ratio of the anteroposterior diameter of the spinal cord to the transverse diameter on an axial slice at maximum compression on T2-weighted sequences (Figure 1).

Four grades were selected:

(A) FR ≥ 0.5;

(B) 0.4 ≤ FR < 0.5;

(C) 0.3 ≤ FR < 0.4;

(D) FR < 0.3.

#### 2.3.2. T2-Weight Intramedullary Hyperintensity

**Figure 2:**
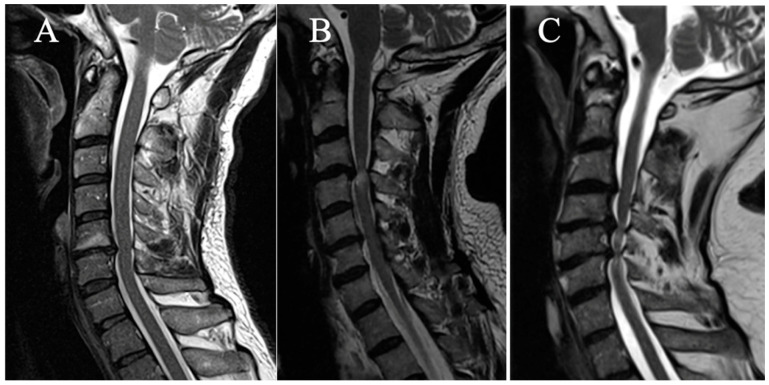
(**A**) No T2HI; (**B**) focal T2HI; (**C**) multifocal T2HI.

Three grades were selected on a sagittal slice (Figure 2):

(A) No T2HI;

(B) Focal T2HI (limited to one intervertebral space and adjacent vertebral bodies);

(C) Multi-segmental T2HI (Extending beyond two intervertebral spaces).

#### 2.3.3. Cerebrospinal Fluid Cisterns

**Figure 3:**
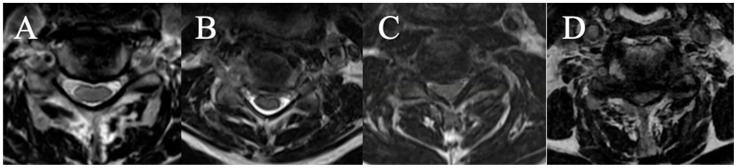
(**A**): CSF visible anteriorly and posteriorly; (**B**) CSF erased anteriorly or posteriorly; (**C**) CSF erased anteriorly and posteriorly but root cisterns visible; (**D**) totally erased cisterns.

Four grades were selected on an axial slice (Figure 3)

(A) CSF visible anteriorly and posteriorly;

(B) CSF erased anteriorly or posteriorly;

(C) CSF erased anteriorly and posteriorly but root cisterns still visible;

(D) Totally erased cisterns—no CSF visible on the slice.

#### 2.3.4. Torg–Pavlov Ratio

**Figure 4:**
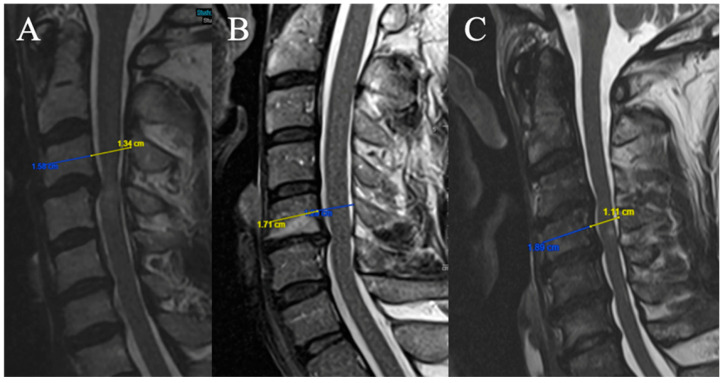
Example of measurement of the TPR. (**A**) TPR = 0.85; (**B**) TPR = 0.75; (**C**) TPR = 0.59.

Three grades were selected (based on T2-weighted sequence on median sagittal sections at the middle vertebral level overlying the compression) (Figure 4). The Torg–Pavlov ratio is calculated using the sagittal canal-to-vertebral body ratio:

(A) TPR ≥ 0.8;

(B) 0.6 ≤ TPR < 0.8;

(C) TPR < 0.6.

#### 2.3.5. Local Kyphosis

**Figure 5:**
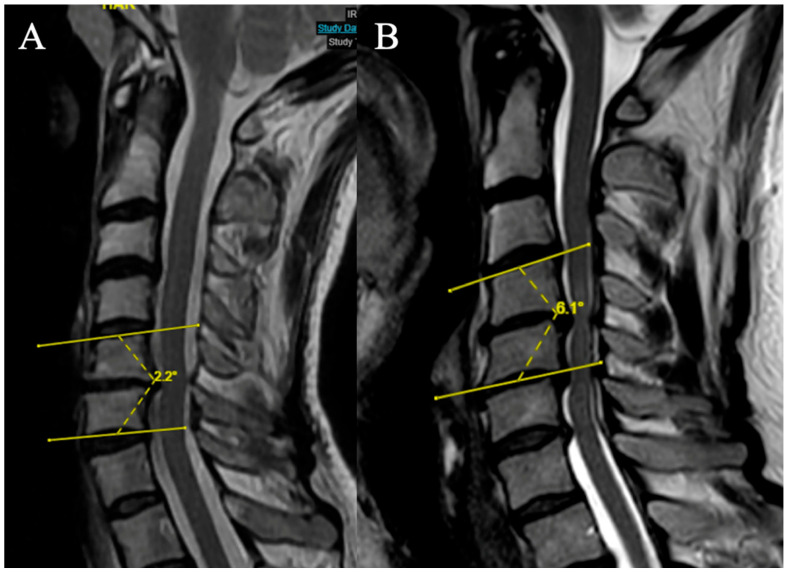
Example of measurement of the local angle. (**A**) LK = 2.2°; (**B**) LK = 6.A°.

Two grades were selected (based on the focal angle measured on sagittal T2-weighted MRI sequences at the level of maximum compression) (Figure 5):

(A) Lordosis or LK <4°

(B) LK ≥ 4°

#### 2.3.6. Number of Stenotic Level(s)

Three grades were selected (any level (in addition to the maximum compression level) for which the FR in the axial section on T2-weighted sequences is < 0.40 is considered as an additional compression level):

(A) One stenotic level;

(B) Two stenotic levels;

(C) ≥Three stenotic levels.

### 2.4. Intraobserver and Interobserver Reliability

Intraobserver reliability was measured by comparing the measurements in 2 cases obtained by two observers (GP and DC) 10 days apart. Conversely, the interobserver reliability was measured by comparing the measurements in 2 cases obtained by the two observers on the same day.

### 2.5. General Statistical Analysis

Quantitative data were described using the mean, standard deviation, median, and first and third quartiles, as well as the minimum and maximum values.

Qualitative data were described using numbers and proportions (percentages).

For the mJOA score, the following 3 categories were used [1]: mild (score ≥ 15), moderate (12 ≤ score ≤ 14), severe (score ≤ 11).

Comparisons of mean mJOA scores for each subgroup of each variable were performed using Student *t* tests.

Associations between quantitative variables were quantified by Spearman’s correlation coefficient, with the associated 95% confidence interval.

Linear regression models were used to assess the association between each of the categorized criteria and the mJOA score. The association was quantified by the coefficient of determination (R2).

Univariate analyses were followed by multivariable regression analyses.

The final choice of criteria to be included in the SIMS score was based on the values of the R2 coefficients obtained in the univariate analysis, on the results of the different multivariate models and on clinical considerations. Conditional on explanatory variables, the mJOA score was normally distributed.

The final association between the selected SIMS and the mJOA score was quantified by the Spearman correlation coefficient (95% confidence interval), and by describing the SIMS score values according to the 3 mJOA categories.

The analyses were performed with the R software.

The intraclass correlation coefficient (ICC) was utilized to measure the intra- and interobserver agreement for different scores, with a confidence interval (CI) of 95%. ICC values of 0.00 to 0.20 were considered to be in slight agreement; 0.21 to 0.40, fair agreement; 0.41 to 0.60, moderate agreement; 0.61 to 0.80, substantial agreement; and 0.81 to 1.00, almost perfect agreement. The analyses were performed with the JASP software ((Version 0.19.1) [Computer software]/https://jasp-stats.org/download/ accessed on 18 September 2024).

## 3. Results

### 3.1. Characteristics of the Cohort (Table 1)

In total, 99 patients met all inclusion criteria, including 41 women (41.4%) and 58 men (58.6%). The mean age at the time of visit was 62.9 years. The minimum age was 28 years, and the maximum was 84 years. The most common level was C5C6 (41.4%). In 17 patients (17.2%), there was no indication for surgery (Table 1).

**Table 1:** Characteristics of the cohort.

	N	%
**Age (yrs)**		
♦ <60	40	40.4
♦ ≥60	59	59.6
**Sex**		
♦ F	41	41.4
♦ M	58	58.6
**FR**	99	
♦ A	8	8.1
♦ B	33	33.3
♦ C	37	37.4
♦ D	21	21.2
**T2HI**	99	
♦ A	44	44.4
♦ B	33	33.3
♦ C	22	22.2
**CSF**	99	
♦ A	6	6.1
♦ B	43	43.4
♦ C	21	21.2
♦ D	29	29.3
**TPR**	99	
♦ A	49	49.5
♦ B	43	43.4
♦ C	7	7.1
**LK**	99	
♦ A	74	74.7
♦ B	25	25.3
**NSL**	99	
♦ A	53	53.5
♦ B	27	27.3
♦ C	19	19.2

The mean mJOA score is 14.9 ± 2.7. The minimum and maximum observed are 7 and 18, respectively. In total, 62 patients (62.6%) presented with mild myelopathy, 22 (22.2%) with moderate myelopathy and 15 (15.2%) with severe myelopathy [2].

### 3.2. Correlation Between Each Variable of the SIMS and mJOA

#### 3.2.1. Fujiwara Ratio

The mean mJOA score was significantly lower for grade C (15.1) compared with grade A (16.9) (*p* < 0.05) and for grade D (12.9) compared with all other grades (Table 2).

**Figure 6:**
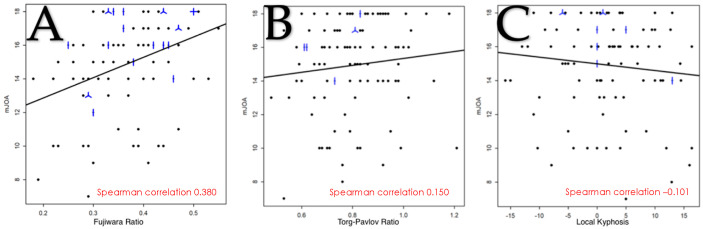
Relationship between the mJOA score and the FR (**A**); the TPR (**B**) and the LK (**C**). A dot represents a single patient, a line 2 patients, a helix 3 patients and a cross 4 patients

The correlation between mJOA score and FR was analyzed using Spearman’s correlation coefficient, and it was 0.380 95% CI [0.20, 0.54] (Figure 6A).

#### 3.2.2. T2-Weight Intramedullary Hyperintensity

The mean mJOA score was significantly lower for grades C (13.1) and B (13.9) than for grade A (16.6) (*p* < 0.05) (Table 2).

#### 3.2.3. CSF Cisterns

The mean mJOA score was significantly lower for all grades compared to less severe grades, with a mean score of 18.0, 16.1, 14.5 and 12.9 for, respectively, grade A, B, C and D (*p* < 0.05) (Table 2).

#### 3.2.4. Torg-Pavlov Ratio

There was no statistically significant difference in the mJOA score between the different grades with, respectively, a score of 15.4, 14.6 and 13.6 (Table 2). The association between TPR and mJOA score was analyzed using Spearman’s correlation coefficient, and was 0.150 95% CI [−0.05, 0.34] (Figure 6B).

#### 3.2.5. Local Kyphosis

There was no statistically significant difference between grades A and B regarding the mean mJOA score (respectively, 15.0 and 14.9) (Table 2).

The relationship between the degree of LK and the mJOA score was analyzed using Spearman’s correlation coefficient, and was −0.101 95% CI [−0.293, 0.098] (Figure 6C).

#### 3.2.6. Number of Stenotic Level(s)

The mean mJOA score for grade B (14.6) was significantly lower than that for grade A (16.0), and the mean mJOA score for grade C (12.4) was significantly lower than that for grade A and B (*p* < 0.05) (Table 2).

### 3.3. Ponderation of the Different Criteria

A linear regression model was used to model the mJOA as a function of the different variables proposed in the SIMS. First, each variable was included separately in the linear model (Table 3). Four variables correlated significantly with the SIMS (*p* < 0.005): FR, CSF, T2HI and NSL.

Secondary, a multivariate linear regression model was created (Table 4). Three variables correlated significantly with the SIMS (*p* < 0.005): CSF, T2HI, and NSL. Unless the FR, TPR or LK did not reach statistical significance, they have been included in the final score for clinical and scientific justification, as developed in the Discussion section.

This model allows us to quantify the importance associated with each grade for each variable and thus to reproduce their relative importance. Indeed, the choice of the points associated with each subscore was made on the basis of the closest unit of the coefficient of determination (except for local kyphosis for which grade B will be given a score of 1 point), calculated as the difference in the mJOA score for each grade compared to the reference grade A (Table 5).

**Figure 7:**
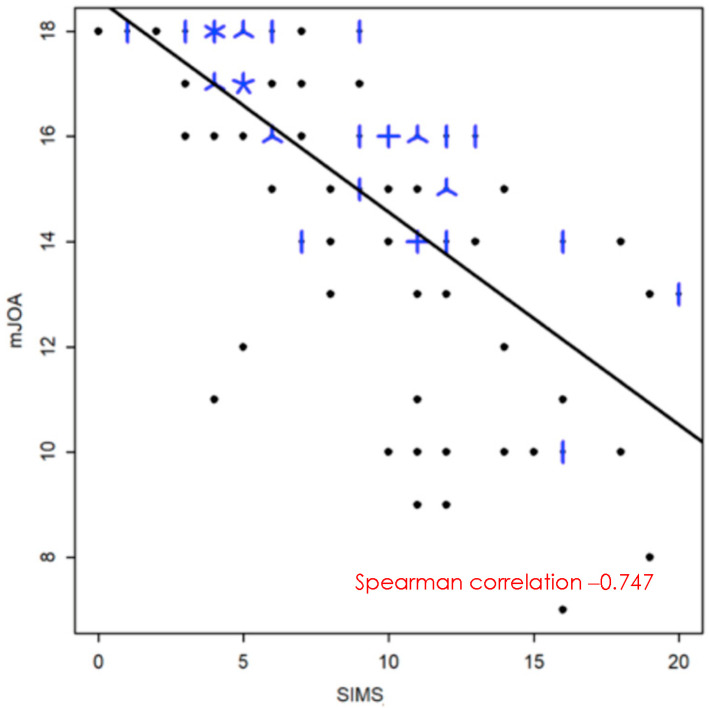
Relationship between the SIMS and the mJOA. A dot represents a single patient, a line 2 patients, a helix 3 patients and a cross 4 patients

Finally, the strength of the relationship between the SIMS and the mJOA score was quantified by a Spearman correlation coefficient of 0.75 (95% CI [−0823, −0.64]) (Figure 7).

### 3.4. Intraobserver and Interobserver Reliability

Table 6 presents the results of the two assessments conducted by the two observers.

Table 7 and Table 8 present the intraobserver reliability and interobserver reliability for both the total score and each individual part, respectively.

Concerning intraobserver reliability, the ICC value was above 0.80, indicating an almost perfect agreement.

Concerning intraobserver reliability, the ICC value was above 0.80, except for the LK, indicating an almost perfect agreement.

## 4. Discussion

At the present time, the most commonly used parameters to assess spinal cord compression in cervical myelopathy on MRI are the appearance of T2HI and the disappearance of peri-medullary fluid spaces. Although T2HI is a marker of disease severity and poor prognosis, it usually occurs at a late stage of the disease [12,13,14,15,16,17].

Some classification systems have been published, such as those of Nagata et al., Muhle et al. or Kang et al., based on obliteration of the subarachnoid spaces, the degree of spinal cord compression or the presence of a change in spinal cord intensity. But these criteria are subjective, evaluate in sagittal plane alone and a correlation with clinical involvement has not been clearly established [18,19,20].

Finally, Wang et al. have also established a new MRI score for assessing compression in patients with posterior longitudinal ligament ossification, following the same model as ours, but without weighting the variables according to their relative importance, and there was no evaluation of the correlation with the mJOA [21].

The choice to retain the T2HI, the CSF and the NSL was natural since they were significantly associated with the JOA score (*p* < 0.001) and are the most useful parameters for routine practice. The results are consistent with those in the literature. For example, Watabe et al. showed that the decrease in CSF flow was significantly associated with the severity of the myelopathy assessed by the JOA [21,22] and Wang et al. showed a significant correlation between different grades of CSF obliteration and the JOA score. Fehlings et al., for their part, found in their prospective surgical cohort an average of 3.86 decompressed levels and showed that the mJOA was inversely proportional to the number of levels affected [2].

In order to be used in clinical routine, MRI parameters must be easily and quickly measurable.

The study of the FR in function of the SIMS showed a significant difference between the different subscores in the univariate modeling but not in the multivariate analysis. The small number of patients with a FR < 0.3 may explain the lack of power for detecting differences between categories. Nevertheless, this variable is easily measurable in routine clinical practice with parameters that have a good interobserver and intraobserver correlation, so it seemed useful to keep in the score [23].

In the same way, the TPR has been integrated into the initial SIMS. Chrispin and Lee found that a ratio of less than 0.85 was a risk factor, which is consistent with the results of Ehni et al. and Pavlov et al. who found ratios of 0.80 and 0.82, respectively [24]. Yue et al. found a limit of 0.72 in their retrospective cohort of 88 patients [25]. Moreover, they demonstrated that in patients over 50 years of age, the relative risk of developing myelopathy was 96% when the Torg–Pavlov ratio was below 0.60. Unfortunately, the small number of patients with a TPR < 0.6 (*n* = 6) in our series does not allow statistical analyses to be performed with correct power, and the fact that TPR was initially described on plain radiographs has to be kept in mind during the interpretation of our results.

Finally, Wu et al. showed that the JOA score had a large and significant negative correlation with focal kyphosis [26]. Oshima et al. found a correlation between segmental kyphosis at the most compressed level and the risk of neurological deterioration requiring surgical conversion in patients with mild degenerative cervical myelopathy [27]. From a mechanical and histological perspective, cervical kyphotic deformity increases the traction force exerted on the spinal cord through stretching of the dentate ligament and nerve roots, as well as causing the spinal cord to be compressed against the vertebral bodies and intervertebral disks. It has been shown that spinal cord flattening, demyelination and atrophy are significantly correlated with the degree of kyphosis and are more pronounced at the apex of the deformity [28,29,30]. The results in this study showed an inverse correlation between the degree of kyphosis and the mJOA, but it was weak and not significant. Nevertheless, given the data in the literature, which show an association of cervical sagittal alignment with the severity of myelopathy and the need to take into account the kyphotic deformity of the cervical spine in the surgical consideration, we decided to keep this parameter in the final score in order to study its relevance more widely in future studies.

The intra- and interobserver reliability of the SIMS are almost perfect, demonstrating that its use could be implemented in routine clinical practice.

The SIMS was strongly correlated with the clinical presentation (evaluated by mJOA) supporting the interest to use a multiparametric score to assess the severity of DCM in clinical practice. Although very encouraging, these results need to be consolidated on a larger, prospective cohort. In addition, interobserver and intraobserver reliability must be analyzed, both between surgeons specializing in the spine and spinal cord, and with other practitioners involved in this pathology (general practitioners, neurologists, rehabilitation specialists, etc.), as this score is intended to be used widely. The second benefit of the prospective evaluation would be to demonstrate the potential prognostic value of this score.

This study had some limitations. First, it was a retrospective study, with some missing data. Then, even if already large with a series of 99 patients, the number of patients could be greater to improve the power of the study, especially concerning moderate and severe myelopathy. Finally, regarding the constitution of the score itself, the maintenance of some variables that were not significantly correlated to the mJOA score on multivariate analyses can be criticized, and their relevance should be confirmed.

## 5. Conclusions

The SIMS scoring system, based on six morphological parameters, represents a coherent and relevant way to characterize the severity of DCM on MRI evaluating the degree of spinal cord compression in sagittal and axial views. The T2-weighted intramedullary change(s), the disappearance of peri-medullary fluid spaces and the number of stenotic levels being the most important factors to be taken into account. It may allow for a standardization of MRI image analyses for DCM and could facilitate comparisons between studies, proving to be a useful evaluation tool.

## Figures and Tables

**Table 2:** Mean mJOA score for each subgroup of variables.

	Mean mJOA ± SD
**FR**	
◆ A	16.9 ± 1.7
◆ B	15.6 ± 2.4
◆ C	15.1 ± 2.6 °
◆ D	12.9 ± 2.6 °*#
**T2HI**	
◆ A	16.6 ± 1.8
◆ B	13.9 ± 2.8 °
◆ C	13.1 ± 2.2 °
**CSF**	
◆ A	18.0 ± 0.0
◆ B	16.1 ± 2.2 °
◆ C	14.5 ± 2.6 °*
◆ D	12.9 ± 2.2 °*#
**TPR**	
◆ A	15.4 ± 2.6
◆ B	14.6 ± 2.8
◆ C	13.6 ± 3.0
**LK**	
◆ A	15.0 ± 2.7
◆ B	14.9 ± 2.7
**NSL**	
◆ A	16.0 ± 2.1
◆ B	14.6 ± 2.3 °
◆ C	12.4 ± 3.1 °*

° compared to grade A (*p* < 0.05); * compared to grade B (*p* < 0.05); # compared to grade C (*p* < 0.05).

**Table 3:** Univariate linear regression model between the variables constituting the SIMS and the mJOA score.

Variable	Grade	Coefficient	Lower 95% CI	UPPER 95% CI	*p*-Value	R2
**FR**	B	−1.495	−3.381	0.391	**<0.001**	0.177
	C	−1.694	−3.563	0.175		
	D	−3.984	−5.982	−1.986		
**T2HI**	B	−2.758	−3.798	−1.717	**<0.001**	0.324
	C	−3.545	−4.725	−2.366		
**CSF**	B	−1.907	−3.881	0.067	**<0.001**	0.329
	C	−3.476	−5.572	−1.380		
	D	−5.138	−7.169	−3.107		
**TPR**	B	−0.802	−1.929	0.325	0.127	0.042
	C	−2.044	−4.362	0.274		
**LK**	B	0.026	−1.296	1.348	0.969	0.000
**NSL**	B	−1.407	−2.534	−0.281	**<0.001**	0.249
	C	−3.579	−4.853	−2.305		

R2: percent of variance explained.

**Table 4:** Results of multivariate linear regression model between the variables constituting the SIMS and the mJOA.

Variable	Grade	Coefficient	Lower 95% CI	Upper 95% CI	*p*-Value
**FR**	B	−0.455	−2.141	1.230	0.725
	C	−0.060	−1.750	1.629	
	D	−0.676	−2.622	1.271	
**T2HI**	B	−1.518	−2.615	−0.420	**0.002**
	C	−2.073	−3.257	−0.889	
**CSF**	B	−1.036	−2.962	0.889	**0.015**
	C	−1.974	−4.260	0.313	
	D	−2.943	−5.228	−0.659	
**TPR**	B	−0.391	−1.285	0.503	0.555
	C	0.379	−1.502	2.260	
**LK**	B	0.695	−0.383	1.773	0.203
**NSL**	B	−0.321	−1.430	0.789	**0.012**
	C	−1.937	−3.266	−0.608	

**Table 5:** The SIMS.

Criteria	Points
Fujiwara ratio	
A. FR ≥ 0.5	0
B. 0.40 ≤ FR < 0.50	1
C. 0.30 ≤ FR < 0.40	3
D. FR < 0.30	4
T2-weighted hyperintensity	
A. No T2-weighted hyperintensity	0
B. Focal T2-weighted hyperintensity	3
C. Multi-segmental T2-weighted hyperintensity	4
CSF	
A. Peri-medullary cisterns visible anteriorly and posteriorly	0
B. Peri-medullary cisterns erased anteriorly or posteriorly	2
C. Peri-medullary cisterns erased anteriorly and posteriorly but root cisterns still visible	3
D. Totally erased cisterns	5
Torg-Pavlov Ratio	
A. TPR ≥ 0.80	0
B. 0.60 ≤ TPR < 0.80	1
C. TPR < 0.60	2
Local kyphosis	
A. Lordosis or LK < 4°	0
B. LK ≥ 4°	1
Number of stenotic level	
A. 1-level	0
B. 2-levels	1
C. ≥3 levels	4

**Table 6:** Measurements of the two examiners.

	Obs1 R1 Case 1	Obs1 R2 Case 1	Obs2 R1 Case 1	Obs2 R2 Case 1	Obs1 R1 Case 2	Obs1 R2 Case 2	Obs2 R1 Case 2	Obs2 R2 Case 2
FR	0.40	0.44	0.43	0.40	0.27	0.21	0.33	0.30
T2IH	0	0	0	0	4	4	4	4
CSF	5	5	5	5	5	5	5	5
TPR	0.75	0.76	0.66	0.69	0.43	0.56	0.53	0.54
LK	7.0	8.9	6.4	9.7	23.5	16.9	1.6	1.1
NSL	0	0	0	0	4	4	4	4
SIMS	7	7	8	8	19	19	19	19

Obs1: observer 1; Obs2: observer 2; R1: first reading; R2: second reading.

**Table 7:** ICC for intraobserver agreement.

	Point Estimate (95% CI)
FR	0.889 (0.539–0.980)
T2IH	1.000 (1.000–1.000)
CSF	1.000 (1.000–1.000)
TPR	0.858 (0.436–0.973)
LK	0.806 (0.290–0.963)
NSL	1.000 (1.000–1.000)
SIMS	1.000 (1.000–1.000)

**Table 8:** ICC for interobserver agreement.

	Point Estimate (95% CI)
FR	0.771 (0.204–0.956)
T2IH	1.000 (1.000–1.000)
CSF	1.000 (1.000–1.000)
TPR	0.813 (0.310–0.965)
LK	0.168 (−0.570–0.778)
NSL	1.000 (1.000–1.000)
SIMS	0.994 (0.971–0.999)

## Data Availability

The original contributions presented in this study are included in the article. Further inquiries can be directed to the corresponding author.

## Abbreviations

The following abbreviations are used in this manuscript:

SIMS	Severity on Imaging Myelopathy Score
DCM	Degenerative Cervical Myelopathy
MRI	Magnetic Resonance Imaging
mJOA	Modified Japanese Orthopaedic Association score
FR	Fujiwara Ratio
T2IH	T2-weighted Intramedullary Hyperintensity
CSF	Cerebrospinal Fluid Cisterns
TPR	Torg–Pavlov Ratio
LK	Local Kyphosis
NSL	Number of Stenotic Levels
ICC	IntraClass Correlation
